# Supplementary material for: A study to evaluate WASH interventions and risk factors of diarrhoea among children under five years, Anloga district, Ghana: A research protocol
Source: PLoS One. 2024 May 24;19(5):e0302754. doi: 10.1371/journal.pone.0302754 (PMC11125463; doi:10.1371/journal.pone.0302754)
Supplement: Attachment — Submitted filename: Review comments.docx [file pone.0302754.s001.docx]

Topic: A study to evaluate WASH interventions and risk factors of diarrhoea among children under five years, Anloga district, Ghana: a research protocol

**Reviewer comments**

**Abstract**

Overall: the Abstract is clear and ok, but some few details are required.

Introduction: You mentioned “decline” – can give us the percentage.

Methodology: Included the analysis that will be done in the evaluation (thematic analysis) and the sampling technique for the part of the research. Include that odds ratio will be obtained at 5% significant level.

Expected outcomes: Let us stop using the word “we”- refer yourself or the people as researchers.

**Introduction**

We need to see more stats in the 1^st^ part of introduction. Let the stats follow from a global perspective to sub-Saharan region to Ghana with regards to childhood diarrhoeal illness.

Also, some stats on WASH intervention should be added in the introduction to give a clear picture why the formulated – connect it to Ghana.

Make your rational or problem statement or justification clear in the introduction because it seems that you are still narrating about diarrhoeal and WASH interventions of the country if there are close to achieving it…etc

Fix some of the grammars and add some references like in Line 10. Line 9-12 – needs to be restructured.

**Methodology**

Aim & Study design: Restructure the first line. Give the rational for using mixed design approach.

Study setting: Fix the first sentence and add the exact location of the study setting (East of Volta region) and add square kilometre of the area. Add a line of the main activities that are done in that region. They can act as the rational for selection of the region as the study site. Mention the communities and am sure they have names. Let the information follow.

Process evaluation: Why did you select qualitative methods (add a rational) and why purposively sampling? Are we interviewing only people that attended the meeting? Make it clear. Just state that …meeting was held on WASH interventions and potential stakeholders that participated will be purposively selected for an interview”.

Characteristics of population and sample selection: Can you justify each sampling method that was used.

Approach 1: Names some stake holders that we are likely to have for evaluation. I think most of information is confusing under some paragraphs. Try and put sub-titles and avoid adding information in wrong sub-titles.

Sampling size calculation: At least give us an estimation of how many participants you like to include by looking at literature.

Sampling: Be clear on the sampling technique and give its rational or since you are doing a lot of sampling methods you can state a multi-stage sampling technique and explain random sampling and purposively sampling. Follow of points is required.

Data collection: Desk reviews (state the data extraction tool will be used) for interviews and FGD (How long will they last, in translation of them??).

Data management & Analysis: State the package that you will be used for analysis for both qualitative and quantitative
